# NG2 cells, a new trail for Alzheimer’s disease mechanisms?

**DOI:** 10.1186/2051-5960-1-7

**Published:** 2013-05-09

**Authors:** Henrietta M Nielsen, Danyal Ek, Una Avdic, Camilla Orbjörn, Oskar Hansson, The Netherlands Brain Bank, Robert Veerhuis, Annemieke JM Rozemuller, Arne Brun, Lennart Minthon, Malin Wennström

**Affiliations:** Department of Clinical Sciences Malmö, Molecular Memory Research Unit, Lund University, The Wallenberg Laboratory 2nd floor, Inga Marie Nilssons gata, entrance 53, Skåne University Hospital, Malmö, 205 02 Sweden; Department of Neuroscience, Mayo Clinic College of Medicine, 4500 San Pablo Road, Jacksonville, FL 32224 USA; Department of Clinical Sciences Malmö, Clinical Memory Research Unit, The Memory Clinic at Skåne University Hospital (SUS), Lund University, Malmö, 205 02 Sweden; The Netherlands Brain Bank, Netherlands Institute for Neuroscience, Amsterdam, The Netherlands; Department of Clinical Chemistry, Neuroscience Campus Amsterdam, VU University Medical Center, Amsterdam, The Netherlands; Department of Pathology, Neuroscience Campus Amsterdam, VU University Medical Center, Amsterdam, The Netherlands; Department of Pathology, Lund University, Lund, 22224 Sweden

**Keywords:** NG2 cells, Alzheimer’s disease, Amyloid beta, Cerebrospinal fluid, Brain tissue, Cell culture

## Abstract

**Background:**

Neuron Glial 2 (NG2) cells are glial cells known to serve as oligodendrocyte progenitors as well as modulators of the neuronal network. Altered NG2 cell morphology and up-regulation as well as increased shedding of the proteoglycan NG2 expressed on the cell surface have been described in rodent models of brain injury. Here we describe alterations in the human NG2 cell population in response to pathological changes characteristic of Alzheimer’s disease (AD).

**Results:**

Immunohistological stainings of postmortem brain specimens from clinically diagnosed and postmortem verified AD patients and non-demented controls revealed reduced NG2 immunoreactivity as well as large numbers of NG2 positive astrocytes in individuals with high amyloid beta plaque load. Since fibrillar amyloid beta (Aβ)1-42 is the major component of AD-related senile plaques, we exposed human NG2 cells to oligomer- and fibril enriched preparations of Aβ1-42. We found that both oligomeric and fibrillar Aβ1-42 induced changes in NG2 cell morphology. Further, *in vitro* exposure to fibrillar Aβ1-42 decreased the NG2 concentrations in both cell lysates and supernatants. Interestingly, we also found significantly decreased levels of soluble NG2 in the cerebrospinal fluid (CSF) from clinically diagnosed AD patients compared to non-demented individuals. Additionally, the CSF NG2 levels were found to significantly correlate with the core AD biomarkers Aß1-42, T-tau and P-tau.

**Conclusion:**

Our results demonstrate major alterations in the NG2 cell population in relation to AD pathology which highlights the NG2 cell population as a new attractive research target in the search for cellular mechanisms associated with AD pathogenesis.

**Electronic supplementary material:**

The online version of this article (doi:10.1186/2051-5960-1-7) contains supplementary material, which is available to authorized users.

## Background

In addition to the three major glial cell types in the brain i.e. astrocytes, microglia and oligodendrocytes, a fourth glial cell type, called the NG2 cell, has been shown to exist. This glial cell is recognized by its stellate morphology and expression of a specific chondroitin sulphate proteoglycan. The proteoglycan is called melanoma cell surface chondroitin sulfate proteoglycan (MSCP) in humans [1], whilst its homologue in rats is named Neuron glia 2 (NG2) [1]. The name NG2 cell has been used to describe the cell type in both species [2]. The NG2 cell has been extensively studied in rats and has been found to constitute the major group of cells undergoing mitosis in the adult rodent brain, representing about 5%–8% of all cells in the nervous system [3, 4]. Although the NG2 cell population was confirmed to exist in the human brain already more than twelve years ago [5], the research field exploring its role within the human healthy and diseased brain is still in its infancy. A possible reason for the previous neglect of NG2 cells as a distinct cell type, may be that NG2 cells can down-regulate NG2 and differentiate into oligodendrocytes [3] and thus have long been considered as a mere progenitor cell. However, many of the NG2 cells remain in the NG2-positive state for a significant time [6], and have a unique capacity to communicate with nearby cells, forming multiple contacts with astrocytes, microglia, oligodendrocytes and neurons [7]. NG2 cells have also been suggested to regulate neuronal signaling, to stabilize synapses and to play a role in the guidance of axonal outgrowth [7].

The chondroitin sulphate proteoglycan NG2 (and its homologue MSPC in humans) expressed on the NG2 cell surface is a type I transmembrane protein with a core glycoprotein of approximately 300 kDa [1]. The extracellular domain contains different cleavage sites and can be shed from the cell surface by proteolytic cleavage. Approximately half of the NG2 proteoglycan in the rodent central nerve system (CNS) belong to the soluble version [8]. The NG2 proteoglycan is not only found on NG2 cells, but also on infiltrating macrophages and on pericytes [9–12].

*In vitro* studies have shown that NG2 cells can become activated, deactivated or differentiate into reactive astrocytes or oligodendrocytes, depending on which factors (including both cytokines and trophic factors) they are exposed to [13]. *In vivo* studies using rodent models have also demonstrated that NG2 cells retract their processes, increase the NG2 expression and start to proliferate in response to severe CNS damage, like stab wounds or epileptic seizures [8, 14, 15]. Moreover, a several-fold increase in soluble NG2 levels has been observed in areas of knife lesioned cerebral rat cortex [8] and in mice spinal cord experimental autoimmune encephalomyelitis (EAE) [16]. These studies indicate a role for the NG2 cell and its proteoglycan in reaction to damage and inflammatory events in the brain. This is supported by the observed loss of NG2 cells and changes in NG2 cell morphology that correlate to disease severity in multiple sclerosis (MS) brain [5, 17, 18]. In the current study we have in detail investigated possible alterations in the human NG2 cell population in response to neuropathology characteristic of Alzheimer’s disease (AD), the most common cause of neurodegenerative dementia.

In AD progressive neurodegeneration leads to cognitive decline manifested as dementia. Accumulation and extracellular deposition of aggregated amyloid-β peptide (Aβ1-42), so called senile plaques, is one of the key events in AD pathogenesis with formation of intraneuronal neurofibrillary tangles (NFTs) of hyperphosphorylated tau and loss of neurons and synapses as downstream events [19]. Correlates to these pathological processes can be monitored in cerebrospinal fluid (CSF), where decreased levels of Aβ1–42 and elevated levels of phosphorylated-tau (P-tau) and total tau (T-tau) are indicative of Aβ1–42 deposition, tangle formation and neurodegeneration respectively [20]. However, not only neurons are affected in AD. As many as 50% of all AD cases also show loss of oligodendrocytes and other white matter components, a condition called white matter disease (WMD) [21, 22]. The underlying cause of oligodendrocyte death has been suggested to include glutamate toxicity [23] and oxidative stress [24] but also toxic effects of Aβ have been reported. Preclinical studies using stereotaxic injection of Aβ1-42 into rat brain have shown that Aβ can induce extensive white matter damage and oligodendrocyte death [25]. In addition, *in vitro* studies on rat oligodendrocytes have demonstrated toxic effects of different Aβ fragments including Aβ1-40 [26], Aβ25-35 [27] and Aβ1-42 [28] as well as the oligomeric form of Aβ1-42 [30]. Other neuropathological features of AD include neuroinflammation and activated astrocytes and microglia are commonly found enwrapping senile plaques in the AD brain. The finding of these so-called glial nests, together with *in vitro* studies demonstrating microglial and astrocytic internalization of Aβ, have led to the conclusion that both astrocytes and microglia play important roles in the clearance and removal of Aβ [30–32].

Thus, AD pathophysiology includes events that theoretically affect the NG2 cell population i.e. brain damage, synapse loss, oligodendrocyte loss and gliosis. In the current study we therefore aimed to investigate the activity and NG2 shedding of human NG2 cells in response to components linked to AD pathology. Using NG2 expressing human primary fetal oligodendrocyte progenitor cells (from here on called NG2^+^HOPC) as a model for human NG2 cells as well as immunohistochemical investigations of postmortem brain tissue and quantification of soluble NG2 levels in CSF from AD patients and non demented controls (ND), we here for the first time describe alterations in the human NG2 cell population in response to AD pathology.

## Results

### NG2 cell characterization in brain tissue from AD patients and non-demented controls

#### Characteristics of post-mortem examined individuals

In order to first investigate whether NG2 cells are affected by AD pathology in the human brain we analyzed NG2 cells in the molecular layer of hippocampus (ML) from clinically and postmortem verified AD patients as well as non-demented (ND) controls. The demographics of the included subjects are given in Table 1. To evaluate AD-related neuropathological changes in the examined individuals we rated the presence of Aβ deposits and microglia nests in the investigated areas (Table 2). As expected, three of the clinically diagnosed AD patient showed extensive plaque load whereas one clinically diagnosed AD patients showed fewer plaques compared to the other AD patients. Two of the ND controls did not exhibit Aβ deposits in ML. Microglial clustering adjacent to plaques, glial nest formation as well as changes in microglial morphology were observed to a higher extent in individuals with high Aβ plaque load (Table 2).

**Table 1:** **Demographics and characteristics of postmortem examined individuals**

NBB (no)	Clinical diagnosis	Gender (M/F)	Age (years)	PMD^a^(hours)	Fixation (hours)	Braak (NFT)	Braak (Aβ)	Braak (LB)
2	AD	F	71	6.26	24	5	C	0
37	AD	F	64	6.44	19	4	C	0
12	AD	F	87	6.42	17	4	C	0
78	AD	M	83	6.22	16	3	B	0
59	ND	F	84	7.65	20	2	B	0
39	ND	F	91	4.15	20	1	B	0
49	ND	F	83	4.40	14	1	B	0
81	ND	M	55	7.30	23	0	0	0
76	ND	F	75	6.75	16	1	0	0

*NFT* = neurofibrillary tangles, *LB* = Lewy body, *AD* = Alzheimer’s disease, *ND* = Non-demented control, *PMD* = post mortem delay.

**Table 2:** **Qualitative assessment of NG2 cell reactivity,** Aβ **plaques and glial nests in post-mortem brain tissue**

NBB (No)	Amyloid plaque (score)^1^	Microglia nest (score)^2^	NG2 cells (IHSI score)^3^	Granular NG2 (%)^4^
AD 2	(++++)	(++++)	(+) **R**	100
AD 37	(+++)	(++++)	(++) **R**	100
AD 12	(++++)	(++++)	(+) **R**	100
AD 78	(++)	(+)	(+++) **R**	50
ND 59	(++)	(++)	(++++) **r**	50
ND 39	(+)	(+)	(+++++) **r**	43
ND 49	(+)	(+++)	(++++)	37
ND 81	**-**	(++)	(+++)	5
ND 76	-	-	(++++)	5

^1^ Plaque load is indicated either as (−) no Aβ plaques visible, or (+) low (++) moderate (+++) high and (++++) very high numbers of Aβ plaques.

^2^ Microglia nests are indicated either as (−) no visible, or (+) low, (++) moderate and (++++) very high numbers of glial nests.

^3^ B5 IHSI intensity of NG2 cells is scored as (−) no visible NG2 cells (+) very weak, (++) weak, (+++) moderate (++++) strong and (+++++) very strong IHSI. R indicates the presence of more than 50% NG2 cells with reactive morphology. r indicate the presence of less than 50% reactive NG2 cells.

^4^ Percentage (%) cells with granular NG2 of total counted cells positive for NG2.stained with 9.2.27 and N143.8.

#### Decreased NG2 immunoreactivity in individuals with AD pathology

**Figure 1:**
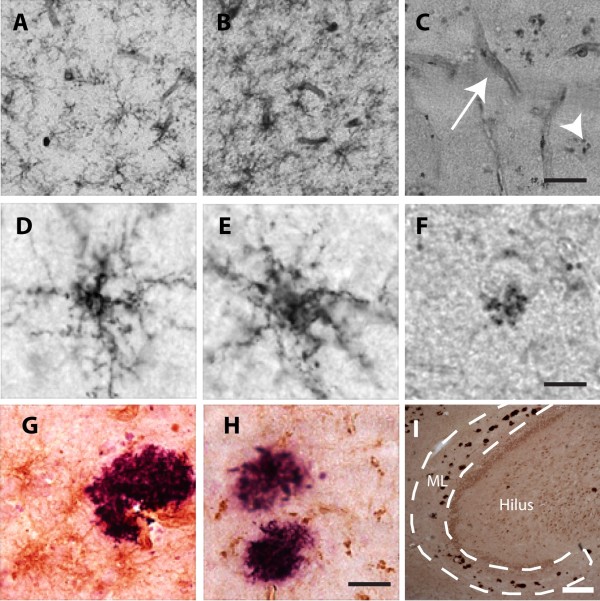
**Microscopic images of NG2 positive cells.** **A**-**C** show low magnification pictures of brain tissue with (**A**) resting NG2 cells, (**B**) reactive NG2 cells and (**C**) granular NG2 (arrowhead) as well as pericytes (arrow). **D-F** show higher magnification images of a resting NG2 cell (**D**), reactive NG2 cell (**E**), granular NG2 (**F**), **G**-**H** show images of reactive NG2 cells (**G**) and granular NG2 (**H**) (brown) adjacent to Aβ plaque (purple), (**I**) shows Aβ deposits in the investigated hippocampal molecular layer (ML) of an AD patient. Scale bar **A**-**C** =150 μm, **D**-**F**=10 μm, **G**, **H**=25 μm and **I**=10 μm.

Next we stained the brain tissue using three different antibodies directed against NG2 and evaluated immunohistological staining intensity (IHSI) as well as morphology of NG2 cells in the ML. The IHSI of NG2 cells varied between individuals and appeared to be dependent on the NG2 antibody used in the staining procedure. All three antibodies gave rise to stainings clearly showing NG2 cells in all ND controls as well as in one of the AD patients) (Table 2). However, the IHSI in the remaining three AD patients were either very weak (when B5 antibody was used (Table 2)) or completely lost (when antibody clones 9.2.27 and N143.8 were used (Figure 1C)). Notably, staining of NG2 on pericytes in these samples was detectable when all three NG2 antibodies were used (Figure 1C). When investigating the NG2 cell morphology we found that NG2 cells in the ML of the two controls without Aβ deposits displayed features of resting NG2 cells i.e. multiple thin, branched processes extending radially from the cell body (Figure 1A and D), whereas most of the analyzed NG2 cells in the AD patients displayed fewer, less branched, shorter processes and swollen cell bodies (Figure 1B and E and Table 3) indicative of a reactive state. Controls with Aβ deposits also showed reactive NG2 cells but to a lesser extent compared to AD patients (Table 2). The NG2 cells were evenly distributed and were not found to cluster around Aβ plaques. The few NG2 cells located nearby plaques were found to display retracted processes (Figure 1G) and in rare cases multi-branched processes (data not shown).

**Table 3:** **Demographic data of individuals with antemortem CSF assessment**

Characteristics	Control (n=36)	AD (n=47)
M/F (n)	17 / 19	13 / 34
Age at investigation (yrs)	61 ± 9	*** 77 ± 7
MMSE	28 ± 1	*** 20 ± 4
APOEϵ4 carriers (%)^1,#^	31	77
Aβ1–42 (ng/L)	659 ± 230	*** 355 ± 100
T-Tau (ng/L)	279 ± 136	*** 821 ± 322
P-Tau (ng/L)^2^	47 ± 19	*** 104 ± 39
NG2 (μg/ml)^3^	1.90 ± 0.07	*1.63 ± 0. 06

Data are presented as mean± SD. ^1^Data missing on n=2 control. ^2^ Data missing on n=2 control, n=10 AD. # Individuals carrying one or two APOEϵ4 alleles. *** Indicates a significant difference at the p<0.001 level compared to controls using ANOVA. ^3^Results are age-adjusted. * Indicates a significant difference at the p<0.05 level compared to controls using ANCOVA.

**Figure 2:**
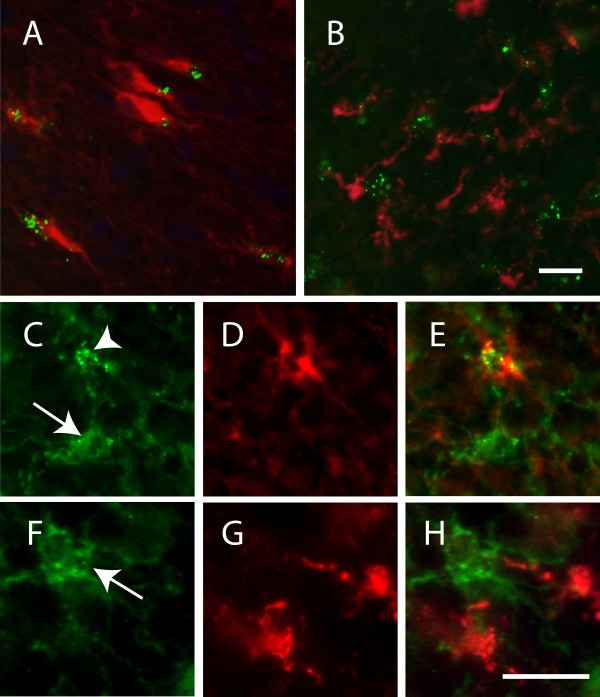
**Images of double staining against NG2 (clone 9.2.27)/GFAP and NG2 (clone 9.2.27)/Iba-1.** (**A**) Co-localisation between GFAP+ astrocyte (red) and granular NG2 (green). (**B**) No co-localisation between iba-1+ microglia (red) and granular NG2 (green). **C-E** represent images of an NG2 and GFAP staining in a higher magnification, where (**C**) shows both ramified (arrow) and clustered (arrowhead) NG2, (**D**) shows a GFAP+ astrocyte and (**E**) a merged picture of the two stainings. **F-G** represent images of NG2 and Iba-1 stainings in a higher magnification where (**F**) shows ramified NG2 cells (arrowhead), (**G**) Iba-1+ microglia and (**H**) a merged picture of the two stainings. Scale bar A, B=20 μm and C-H=15 μm.

Interestingly, staining using the antibody clones 9.2.27 and N143.8, but not B5, revealed the presence of cells displaying granular dense aggregates of NG2 (from here on called granular NG2) (Figure 1F and Additional file 1: Figure S3). Cells with granular NG2 were found in both AD patients and ND individuals and the percentage cells with NG2 of total cells positive for NG2, increased with Aβ plaque load (Table 2). Further, double immunofluorescence staining against NG2 and microglia marker Iba-1 showed that none of the resting or reactive NG2 cells or cell with granular NG2 were positive for Iba-1 (Figure 2B, 2F-H). However, staining against NG2 (9.2.27 or N143.8) and the astrocytic intrafilament (glial fibrillary acidic protein (GFAP)) revealed a close co-localization of granular NG2 with GFAP (Figure 2A, 2C-E and Additional file 1: Figure S3) and the granular NG2 were often found on one side of the astrocyte. Neither resting nor reactive NG2 cells were positive for GFAP (Figure 2C-E).

### In vitro characterization of NG2^+^HOPC responses to Aβ

#### In vitro NG2^+^HOPC morphology is altered in response to Aβ1–42

**Figure 3:**
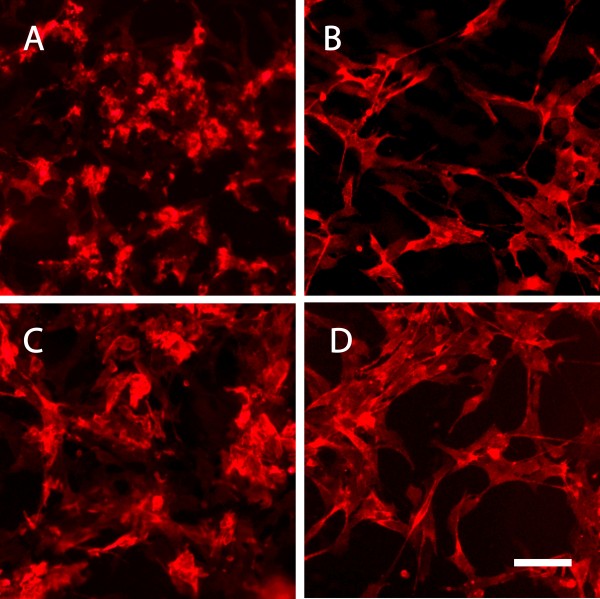
**Fluorescence micrographs of NG2+HOPCs stained for NG2.** Images show **A**) NG2+HOPCs after 18 h exposure to 10 μM Aβ1–42 oligomers. **B**) NG2+HOPCs after 18 h exposure to Aβ1–42 oligomer vehicle. **C**) NG2+HOPCs after 18 h exposure to 10 μM Aβ1–42 fibrils. **D**) NG2+HOPCs after 18 h exposure to Aβ1–42 fibril vehicle Scale bar = 25 μm.

Our postmortem examination revealed changes in NG2 cell morphology in brain areas with high Aβ plaque load. Since Aβ1–42 is the major component of senile plaques [20] we aimed to investigate how NG2^+^HOPC react upon *in vitro* exposure to different aggregations forms of Aβ1–42. Upon exposure to Aβ1–42 oligomer enriched preparations, NG2^+^HOPC retracted their processes and the cell bodies appeared dense (Figure 3A) compared to cells treated with oligomer vehicle (Figure 3B). Similar changes were detected also after Aβ1–42 fibril exposure, slight retraction of processes and more compact cell bodies (Figure 3C) compared to cells treated with fibril vehicle (Figure 3D), however not to the same extent as seen after Aβ1–42 oligomer exposure.

#### In vitro NG2 concentrations in NG2+HOPC cell lysates and supernatants decrease in response to Aβ1–42

**Figure 4:**
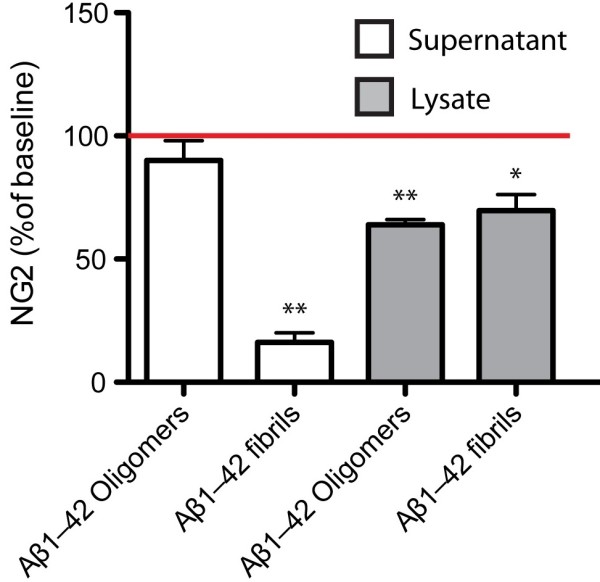
**Changes in NG2 levels (% of vehicle) in cell culture supernatants and cell lysates after exposure to 10**μ**M Aβ1–42 oligomers and 10** **μM Aβ1–42 fibrils for 18 h.** Each bar represents the mean ± SEM of 3 independent experiments. Data was analyzed with paired t-test. * indicates a significant difference at p< 0.05 level compared to baseline ** indicates a significant difference at p< 0.01 level compared to baseline.

Results obtained by use of the NG2 enzyme-linked immunosorbent assay (ELISA) showed significantly reduced cell lysate NG2 levels after exposure to both Aβ1–42 oligomers and fibrils compared to lysates from vehicle exposed cells (61.86 ± 2.34 μg/ml vs. 42.95 ± 3.69 μg/ml, p=0.023 and 56.18 ± 3.69 μg/ml vs 35.87 ± 1.945 μg/ml, p=0.001). Further, a decrease in NG2 levels was found in cell supernatants from cells exposed Aβ1–42 fibrils compared to vehicle-treated cells (0.21± 0.090 μg/ml vs 1.29 ± 0.29 μg/ml, p=0.06) (Figure 4).

#### 24 h exposure to Aβ1–42 does not alter NG2+HOPC viability

To evaluate potential cytotoxic effects of Aβ1–42 oligomers and fibrils on NG2+HOPCs, we measured cell viability as a function of lactate dehydrogenase (LDH) activity in the cell culture supernatants. As expected, cells exposed to the positive control Staurosporine exhibited significantly increased extracellular LDH activity compared to untreated cells (256.42% more, p<0.001). No changes were seen after exposure to either Aβ1–42 oligomers or Aβ1–42 fibrils.

### Soluble NG2 concentrations in cerebrospinal fluid from AD patients versus non-demented controls

To investigate if the observed *in vitro* impact of Aβ1-42 on levels of soluble NG2 is reflected in CSF, we analyzed CSF from AD patients (n=47) and ND (n=36). The demographic data, MMSE scores, APOEϵ4 allele frequency and CSF levels of the well-established AD biomarkers are found in Table 3. As expected, AD patients had significantly lower MMSE scores (p<0.001) and a higher frequency of one or two APOE4 alleles compared to controls (Table 3). Concentrations of Aβ1–42 were significantly lower (p<0.001), whereas P-tau (p<0.001) and T-tau (p<0.001) were significantly higher in AD patients compared to controls. The AD patient group was significantly older than the control (p<0.001).

#### Levels of NG2 are decreased in CSF from AD patients

Since the ND individuals were significantly younger than the AD patients, we first evaluated whether NG2 CSF levels were associated with age in the group of ND by use of correlation analysis. We found a significant correlation between CSF NG2 levels and age in the ND group (r = 0.532, p=0.001) as well as in AD patients (r=0.342, p=0.19) and thus age was added as a covariant in the statistical analyses. Comparison analysis showed that AD patients had significantly lower levels of NG2 in the CSF compared to controls (ANCOVA p=0.010). Graph of NG2 levels, unadjusted for age, are found in Additional file 2: Figure S4.

#### Levels of NG2 in CSF correlate with CSF AD biomarkers

To investigate potential associations between NG2 concentrations, cognitive decline (mini–mental state examination (MMSE)) as well as the AD CSF biomarkers Aβ1–42, T-tau and P-Tau, we used partial correlation analysis with age as a covariant. We found a correlation between NG2 levels and Aβ1–42 levels in the AD patients group (r=0.406, p=0.005). A significant correlation was also found between P-tau, and NG2 in the control group (r=0.396, p=0.022) and the AD group (r=0.424, p=0.01) and between T-tau and NG2 in the control group (r=0.345, p=0.042) as well as in the AD group (r=0.352, p=0.016). No links between NG2 levels and cognitive decline was found in either group and no significant change in NG2 levels were found when comparing males / females or APOEϵ4 carriers/non-carriers within the analyzed groups (data not shown). Plotted graphs of the unadjusted correlations between NG2 levels and Aβ1–42, T-tau and P-tau are found in Additional file 2: Figure S4.

## Discussion

In the current study we show that individuals with high Aβ plaque load display reduced NG2 immunoreactivity as well as a high frequency of NG2 positive astrocytes in the molecular layer of hippocampus. We also found a significant aggregation-state dependent Aβ1–42 effect on morphology and NG2 expression/shedding of cultured primary human NG2+HOPC. Further we quantified the levels of soluble NG2 in the CSF of AD patients and the ND group and showed that NG2 levels increased with age and that AD patients displayed lower CSF NG2 levels compared to ND. Last, we found significant associations between the well-established AD core biomarkers and NG2 CSF levels. Taken together our results clearly demonstrate an impact of AD pathology on NG2 cell activity and NG2 expression/shedding.

In line with previous studies of brain tissue from rodents exposed to neuroinflammatory stimuli (such as stab wounds and epileptic seizures [8, 14, 15]) and patients with different pathologies (including epilepsy, global cerebral ischemia, sepsis and cerebral hemorrhage) [2], we found reactive NG2 cells in individuals with AD pathology. Given our *in vitro* findings showing morphological changes after both Aβ1-42 oligomer and fibril exposure, it is tempting to speculate that the described changes in NG2 cell morphology in brain tissue might reflect prolonged exposure to high Aβ1-42 load. However, although Aβ1-42 to some extent may affect the cell morphology we cannot exclude the possibility that the observed changes could be due to the neuroinflammatory events linked to AD pathology. As in previous studies of brain tissue from AD patients [33], we found strong glial activation and presence of glial nests in individuals with AD pathology. Moreover glial nests increased in number with pathology severity. The coexistence of reactive microglia and reactive NG2 cells has been described in both clinical and preclinical studies [2, 34] and NG2 cells are often found spatially close to microglia [34]. In addition, *in vitro* studies have shown that NG2 cells strongly respond to factors secreted by activated microglia [35–37] and it has been suggested that the NG2 activation, induced by microglial activity, occurs at the expense of NG2 cell differentiation into mature oligodendrocytes [38, 39]. Thus, it is possible that the changed NG2 cell morphology might reflect a change in cell activation related to AD pathology-induced neuroinflammation and microglial activation.

Previous studies have also shown that systemically administered lipopolysaccharides (LPS) induce expression of NG2 by microglia [40]. We therefore investigated potential overlaps between NG2 immunoreactivity and the microglial specific marker (Iba-1) and found no co-expression of these two antigens. Based on the lack of NG2 and Iba-1 overlap, the morphology of the NG2 positive cells (bipolar cell bodies with spine-like perpendicularly branched processes) as well as the difference in distribution of the two cell types around amyloid plaques, we conclude that the observed NG2 immunoreactive cells are indeed NG2 cells.

The IHSI of NG2 in three out of four investigated brains from AD patients was very weak (B5 staining) or undetectable (9.2.27 and N143.8 staining). Previous studies have shown that postmortem delay (PMD) and post fixation duration can affect NG2 immunoreactivity. In the current study, however, the PMD and post fixation duration did not differ between AD and ND. Also, due to the fact that NG2 expressing pericytes were indeed visible in tissue from these AD patient patients, we would like to argue that tissue handling was not the underlying cause of NG2 immunoreactivity loss. Whether absence of visible NG2 cells resulting from NG2 cell death, differentiation or merely a reduction of detectable NG2 remains to be investigated, but our *in vitro* result demonstrating a reduction in levels of NG2 in both supernatants and lysates of NG2+ HOPC cells in the presence of Aβ1-42 are in line with the latter hypothesis.

Interestingly, we also observed cells with NG2 immunoreactivity appearing as aggregated NG2 granules frequently located on one side of the cell nuclei. The number of these cells appeared to increase with AD pathology severity and was detected using two different NG2 antibody clones, 9.2.27 and N143.8, whereas B5 failed to capture granular NG2. Although, the epitope for N143.8 is known to be located at the membrane proximal domain 3 of the NG2 core protein [41], less is known about the epitope location for clone B5 and 9.2.27. However, our study suggests that the granular NG2 represents fragments of NG2 displaying epitopes only for clones 9.2.27 and N143.8.

As described before, we found no co-labeling between the microglia marker Iba-1 and granular NG2, however staining against the astrocytic intracellular filament GFAP showed that the large majority of granular NG2 co-localized with GFAP. The co-localization could theoretically be due to NG2 cell differentiation into astrocytes, astrocytes temporarily expressing NG2, astrocytic cell surface binding to soluble NG2 or astrocytic NG2 uptake. Indeed there are several rodent studies suggesting that NG2 cells can generate reactive astrocytes at sites of CNS injury [42–46]. However, the fact that the NG2 granules in our study appeared in clusters located in restricted areas close to the intrafilament GFAP and not evenly spread out on the surface of the astrocytes, speaks in favor of phagocytic activity.

In response to injury NG2 cells not only change morphology, they also upregulate the expression of NG2 and the amount of soluble NG2 shed into the surroundings increases. For instance, several-fold higher levels of soluble NG2 can be observed in the rat cortex after knife lesions [8]. Interestingly, we here found that both oligomer and fibril enriched preparations of Aβ1-42 instead reduced levels of NG2 in NG2+HOPC cell lysates and that fibrillar, but not oligomeric Aβ1-42, reduced NG2 levels in cell culture supernatants. Since we found no significant difference in NG2 cell viability upon exposure to either Aβ1-42 oligomeric or Aβ1-42 fibrillar preparations, a finding in line with a previous study proposing an OPC relative resistance to Aβ1-42 toxicity [29], we suggest that the reduced levels of NG2 are caused by an aggregation-dependent direct or indirect effect of Aβ1-42 on shedding and expression, possibly involving both inhibition of enzymatic shedding and downregulation of NG2 expression.

The significance of the impact of Aβ1-42 on NG2 levels remains elusive, as literature describing the exact function of NG2 is scarce. However, lessons learned from studies within the field of glioma demonstrate that NG2 cells have the ability to potentiate or regulate the activities of integrins or growth factor receptors and thereby play an important role in NG2 cell motility and proliferation [47]. Further, results from *in vivo* studies suggest an important role of NG2 in neurite outgrowth [48] and NG2 has been proposed to be involved in the formation of synapses between NG2 cells and neurons [49]. It should be noted that our *in vitro* studies were performed on cells isolated from fetal white matter. Thus, although the literature suggests that the perinatal OPCs react to the same stimuli as postnatal OPC [50] previous studies have shown differences in certain properties such as proliferation rate and differentiation fate [51]. Further, some studies have suggested that the NG2 cell population is heterogeneous [52]. We should therefore bear in mind that cells from different brain areas may respond differently to Aβ exposure.

To investigate whether alterations in NG2 shedding can be monitored clinically, we analyzed NG2 concentrations in CSF from AD patients and ND individuals. Our results showed that CSF levels of NG2 in both AD patients and non-demented controls increased with age. Although a link between NG2 expression and age has not been properly investigated before, previous studies on rats have demonstrated age-related changes in NG2 cell proliferation and differentiation. From birth and up until middle age NG2 cells were shown to proliferate yielding an increase in the NG2 cell population. With increasing age, proliferation declines, but so does the differentiation of NG2 cells into mature oligodendrocytes [53]. A recent study on glial cells in the cerebral cortex from aging primates instead suggested that the number of oligodendrocytes increase with age and that the increase is most probably due to NG2 cell proliferation [54]. Another plausible explanation could be linked to changes in NG2 cell activity. Microglial activation increases with age and given the previously discussed co-activation of these cell types, it is likely that NG2 cells also increase their activation and thereby increase the NG2 expression.

Our analysis further demonstrated that AD patients displayed significantly lower NG2 levels in the CSF compared to healthy elders when age was used as a co-variant. Whether the observed reduction in NG2 levels is due to reduced expression, shedding, clearance or even loss of NG2 cells due to cell death or inhibited proliferation is yet to be determined. Notably, the observed reduction is in line with the results from our *in vitro* studies showing a reduction in NG2 concentrations in response to Aβ1-42 exposure. Moreover, recent preliminary work by Honeywell and colleagues demonstrated that a decrease in the NG2 cell population as well as reduced NG2 levels in the 3xTg-AD mouse model coincide with increased Aβ levels in brain (abstract published in Neuroscience Meeting Planner No. 352.15/W8. Washington, DC: Society for Neuroscience, 2011). Importantly, we also found a positive correlation between the CSF levels of Aβ1-42 and NG2 levels in the investigated AD patients. Since decreased levels of Aβ1-42 in CSF have been shown to correlate with increased Aβ load in the brain [55], we suggest that the positive correlation implies an association between lower Aβ1-42 plaque load (thus higher CSF Aβ1-42) and higher NG2 levels, and vice versa, further supporting the hypothesis that Aβ affects the amount of soluble NG2. Finally, there is to date no way to determine the cellular origin of the NG2 levels quantified in the CSF, which complicates the interpretation of reduced CSF NG2 levels as a result of AD-pathology-related effects on specific NG2 expressing cell types.

## Conclusion

Our described results suggest that the NG2 cell population is strongly affected by components involved in AD pathology. The significance of our findings is underlined by an increasing number of studies describing NG2 cells as regulating cells, which tightly communicate with the neuronal network. Previous studies have also demonstrated changes in the NG2 cell population in response to various stimuli known to affect emotional processing and spatial memory including stress hormones [56], electroconvulsive seizure (rat model for the antidepressant treatment electroconvulsive therapy) [56–58], lithium [59] and enriched environment [60]. Moreover, the role of NG2 cell as oligodendrocyte progenitors, differentiating when re-myelination is needed [30, 61], inevitably leads to the speculation that alterations in this population pool or even a failure of differentiation due to microglial activation [38, 39] could potentially in part underlie the oligodendrocyte loss and white matter damage found in AD patients. We therefore conclude that our current study, a first step to investigate the activation state and NG2 expression/shedding of human NG2 cells in the presence of AD pathology, highlights the NG2 cell population as an attractive and still rather unexplored research target in the search for cellular mechanisms related to AD pathogenesis.

## Methods

### Postmortem brain tissue studies

#### Brain tissue

Postmortem human brain tissue was obtained from the Netherlands Brain Bank (NBB) (project nr 2010/636). Written informed consent for the use of brain tissue and clinical data for research purpose was obtained from all patients or their next of kin. The study included n=4 clinically and postmortem verified AD patients and n=5 ND [62] (see Table 1). One cm thick slices from the midlevel of hippocampus entorhinal cortex were dissected at autopsy within 7 hours postmortem. The tissue was post fixed in 4% paraformaldehyde for 14–20 hours and left in phosphate buffered saline (PBS) containing 30% sucrose for 4 days. Sections, 30μm thick, were cut using a cryostate and stored free floating in antifreeze cryoprotectant solution at −20°C until analyzed using immunohistochemistry.

#### Immunohistological stainings

Brain tissue sections were analyzed with immunohistological techniques using the mouse polyclonal anti-NG2 (1:1, clone B5, ATCC, kind gift from Dr William Stallcup, ATCC), mouse monoclonal anti-NG2 (1:200, clone 9.2.27, Millipore), mouse monoclonal anti-NG2 (1:200, clone N143.8, kind gift from Dr William Stallcup) mouse monoclonal anti-Aβ1-17 (1:400, clone 6E10, BioSite), rabbit anti-Iba-1 (1:200, Wako) against actin-linking molecule expressed by macrophages and microglia [63].

Tissue sections were quenched in 3% H_2_O_2_ and 10% methanol for 30 min and incubated in Impress reagent kit blocking solution (Vector laboratories) for 1 h at RT, followed by incubation with primary antibodies (anti-NG2, anti Aβ-1-17, anti-Iba-1) in blocking solution overnight at 4°C. Sections were thereafter incubated with appropriate (i.e. either anti-rabbit Igs or anti-mouse Igs) Impress reagent kit secondary antibodies (Vector Laboratories) for 2 h at RT followed by peroxidase detection for 3 min (0.25 mg/ml diaminobenzidine with 0.028% NiCl and 0.012% H2O2 (brown color) or Nova red (red color) or VIP (purple color) (Vector Laboratories). Sections were mounted, dehydrated and cover-slipped. Three sections from each staining and subject were analyzed with an Olympus AX70 light microscope equipped with 4 - 40× objectives. The percentage of cells with granular NG2 of total number of counted NG2+ cells (approximately 200 cells) in ML was calculated. Activity state of NG2 cells were estimated based on previously described changes in NG2 cell morphology (swollen cellbodies, retracted processes and increased NG2 immunoreactivity) [14]. Number of reactive and resting NG2 cells were estimated and ranked as (R) indicating more than 50% reactive NG2 cells or (r) indicating less than 50% reactive NG2 cells. Two independent blinded observers semi-quantitatively scored the intensity of the immunohistochemical staining (IHSI) of NG2 as: (−) no visible NG2 cells, (+) weak (++) moderate, (+++) high and (++++) very high IHSI (see Table 2). Amyloid-beta deposits were semi-quantitatively scored as: (−) no visible, (+) low number, (++) moderate number and (++++) very high number of Aβ plaques (see Table 2). Presence of microglial nests was also semi-quantitatively scored as: (−) no visible, (+) low number, (++) moderate number and (++++) very high number of microglia nests (see Table 2).

#### Double immunoflouresence stainings

Tissue sections were incubated with blocking solution (5% goat serum (Jackson immunoresearch) and 5% human serum (in house)) for 1 h at RT followed by incubation with mouse anti-NG2 (B5, 9.2.27 and N143.8) together with either rabbit anti-Iba-1 (1:200, Wako) or rabbit polyclonal anti-Glial fibrillary acidic protein (GFAP) (1:400 Dako) in blocking solution overnight at 4°C. Sections were incubated with goat anti-rabbit Dylight 549 (1:500, Vector laboratories) and goat anti-mouse Alexa488 (1:500, Invitrogen) and mounted with Vectashield Set mounting medium containing DAPI (Vector laboratories). Sections were analyzed using confocal microscopy.

### In vitro NG2^+^HOPC studies

#### Cells

Fetal primary human oligodendrocyte precursor cells (HOPCs) (Sciencell Research Laboratories) were cultured in cell culture medium (Sciencell Research Laboratories) supplemented with 10% fetal bovine serum (Gibco), as recommended by the supplier. Cells were grown as monolayers in poly-L-lysine (PLL) coated culture flasks in humified air with 5% CO_2_ at 37°C until 80–90% confluent. Prior to experiments the cells were harvested by gentle scraping, plated in 12 well poly-L-lysine coated plates (Gibco) and grown until 70% confluent.

#### Preparation of Aβ oligomer- and fibril enriched preparations

Oligomer and fibril enriched Aβ1-42 preparations were generated as previously described [30]. Differences in Aβ1–42 oligomer and Aβ1–42 fibril constituents of the two preparations, prior to and upon treatment completion, were recorded using western blot (WB) as described before [30] (Additional file 3: Figure S1).

#### NG2^+^HOPC treatment

For cell treatment the culture media was removed and replaced with serum-free media (Sciencell Research laboratories) containing 10μM oligomeric Aβ1-42 or 10 μM fibrillar Aβ1-42. Cells exposed to DMSO/phenol red free DMEM (oligomer vehicle) or DMSO/ HCl (fibril vehicle) were used as baseline controls. Cell treatment was conducted for 18 h at 37°C in 5% CO_2_, Experiments were performed in duplicates and repeated independently three times. After treatment the cell culture supernatants were collected, centrifuged (275× g, 5 min, 4°C), aliquoted and stored at −80°C until used. The cells were lysed with mammalian Cell Lysis kit (Sigma-Aldrich) according to manufacturers’ protocol, aliquoted and stored at −80°C until used. Similar protein concentrations in the wells with different treatment conditions were determined by the use of the Bradford (coomassie plus) assay kit (Thermo scientific).

#### Immunocytoflourescence stainings of NG2^+^HOPC cultures

Cell culture purity was investigated by staining untreated cells with mouse anti-NG2 and rabbit anti-PDGF AA receptor (1:200, Abcam). To investigate morphological changes upon Aβ1-42 challenge the cells were incubated with 10 μM oligomeric Aβ1-42 and 10 μM fibrillar Aβ1-42, and corresponding vehicles for 18 h at 37°C and thereafter stained with mouse anti-NG2. Each condition was applied in duplicate and the experiments repeated independently three times. Staining procedures were as follows: Cells were fixed with 4% formaldehyde, incubated with blocking solution (PBS containing 1% BSA (Boehringer Mannheim) and 5% goat serum (Jackson immunoresearch)) followed by incubations with the primary antibodies (anti-NG2 and anti-PDGFAAR in blocking solution) and the secondary antibodies (Alexa 488-conjugated anti-rabbit Igs (1:500 Molecular probes) and/or Cy3-conjugated anti-mouse Igs (1:500, Jackson Immunoresearch)), Cells were mounted with Vectashield Set mounting medium with DAPI (Vector laboratories). Approximately 94% of the cultured cells (n=200 counted) expressed NG2. The percentage of PDGF-AAR positive cells (n=200 counted) was 92%.

### NG2 quantification assay

The antibody used in our in house developed assay for NG2 quantification has previously been shown to recognize both rat and human NG2 [64–66]. To further evaluate the antibody we performed immunoprecipitation and western blot of concentrated (speed-vac concentrator system), enzymatically digested or undigested (0.025 U/ml Chondroitinase ABC (Seikagaku) lysates and culture supernatants of NG2+HOPC cells as well as pooled human CSF. The immunoprecipitation Kit (Sigma-Aldrich) was used according to the manufacturer’s instructions. Mouse IgG was used as a non-relevant negative control. The precipitated samples were separated using 3-8% tris-acetate gels (Invitrogen) under reducing conditions. Proteins were transferred to membranes (Highbond ECL, GE healthcare) using a trans-blot semi-dry transfer cell (Bio-Rad) at RT for 2.5 hours at 50mA per gel. The membranes were blocked with 5% skimmed milk in 0.05 PBS-T (Fluka analytical) for 1 h and probed with B5 anti-NG2 antibodies in blocking solution overnight at 4°C. The membranes were then incubated with biotinylated rabbit anti-mouse (1:2000 Dako) in blocking solution for 2 h at RT followed by incubation with high-sensitive streptavidin (1:4000, Pierce) for 1 h RT. A smear at approximately 290–400 kDa in wells loaded with undigested lysates and supernatants from NG2+HOPC as well as the presence of distinct bands at 300–240 kDa in wells loaded with digested CSF and NG2+HOPC lysates and supernatants were in line with previous studies [9, 41, 67, 68] and confirmed the specificity of B5 against the NG2 proteoglycan (see Additional file 4: Figure S2).

The in-house assay for NG2 quantification was developed using the Mesoscale Discovery (MSD) electrochemiluminescence (ECL) technology employing immunoassay conversion kits (Mesoscale Discovery). Recombinant rat NG2 protein [69] and samples (NG2^+^HOPC cell culture supernatants and lysates, and human CSF) were diluted in PBS and coated onto MSD multi array plates in duplicate wells (25μl) and allowed to adhere overnight at 4°C. After rinsing, wells were incubated with blocking solution (PBS-T containing 1% BSA and 1% skimmed milk) for 1 h at RT. After another rinse the wells were incubated with mouse anti-NG2 (B5) for 2 h at RT on an orbital shaker, followed by rinsing and incubation with sulfo-Tag goat-anti mouse Igs (Mesoscale) for 1 h in RT on an orbital shaker. Resulting ECL signal was quantified using an MSD SECTOR Imager 6000. Signal from wells incubated with CSF and sulfo-Tag anti-mouse Igs (secondary antibodies) did not differ from zero standard indicating no antibody-related unspecific binding. Readings of the duplicate standards and samples were averaged and NG2 concentrations determined by interpolation of a 4 parametric curve fit. The intra- and inter-assay variation coefficients (CV%) were <10 and <23 and recovery of spiked standards (recombinant rat NG2) into CSF was between 92–107%. To limit the effect of inter-assay variations two controls samples were included in each run to determine the inter-assay variability. Detection limit was determined to be 58.81 ng/ml.

#### Cytotoxicity assay

Cell viability was determined as a measure of lactate dehydrogenase activity (LDH) in the NG2^+^HOPC cell culture supernatants as previously described [70]. Cell culture supernatants from NG2^+^HOPC cells treated with dimetylsulfoxid (DMSO)/phenol red free Dulbecco’s Modified Eagle Medium (DMEM) (Aβ oligomer vehicle) and DMSO/ HCL (Aβ fibril vehicles) were used as negative controls and supernatants from cells treated 18 h with 1 μM Staurosporine (Sigma-Aldrich) were used as a positive control for cytotoxicity. Resulting K values were averaged (± SD) into percentage activity of corresponding negative controls.

### Cerebrospinal fluid studies

#### Patients

The studied groups consisted of n=47 clinically diagnosed AD patients and n=36 ND evaluated at the Memory Clinic at Skåne University Hospital, Malmö, Sweden. Clinical diagnoses were set according to the Diagnostic and Statistical Manual of Mental Disorders by the American Psychiatric Association (DSM-IV, 1994) combined with NINCDS-ADRDA diagnostic criteria [71] for probable AD. Cognitive status of patients and controls was evaluated using the Mini Mental State Examination [72]. The CSF AD-biomarkers (Aβ1-42, T-tau, P-tau181) were analyzed in clinical routine by commercial ELISA kits (Innogenetics). The study protocol was approved by local ethics committee of Lund University and conducted in compliance with the Helsinki declaration of 1975 (revised in 2000). All individuals gave informed consent to participate in the study.

#### Statistical analysis

Statistical analysis was performed using the SPSS software (version 20.0 for Windows, SPSS Inc., Chicago, IL, USA). The Kolmogorov–Smirnov test was used to assess normal distribution and the paired samples t-test was used to detect significant differences in response to *in vitro* cell treatment. Differences in CSF NG2 concentrations were assessed by the use of ANCOVA (controlling for age), followed by the LSD post-hoc test. Correlations between the investigated variables were examined using the Pearson correlation test or partial correlation test (controlling for age). Results are presented as means ± standard deviation. A p < 0.05 was considered significant.

## Electronic supplementary material

Additional file 1: Figure S3: Confocal images showing the hippocampal molecular layer of an AD patient double stained with the three different NG2 antibody clones B5, 9.2.27 and N143.8 (green) together with GFAP (red). Merged images of the respective NG2 antibody and GFAP are found in the lane far to the right. Scale bar = 25 μm. (TIFF 18 MB)

Additional file 2: Figure S4: Plots showing **A**) Unadjusted NG2 levels in CSF from AD patients and non-demented controls prior to ANCOVA analysis with age as a covariant (AD=Patients with Alzheimers’s disease, Ctrl = non-demented controls). **B**) NG2 levels in CSF from non-demented controls correlates with age (p=0.001). **C**) Unadjusted NG2 levels and Aß1–42 levels in CSF from AD patients prior to partial correlation analysis with age as a covariant. **D**) Unadjusted NG2 levels and Total tau (T-Tau) levels in CSF from AD patients prior to partial correlation analysis with age as a covariant. **E**) Unadjusted NG2 levels and hyper-phosphorylated tau (P-Tau) levels in CSF from AD patients prior to partial correlation analysis with age as a covariant. (TIFF 8 MB)

Additional file 3: Figure S1: Oligomer and fibril preparations were incubated for 24 h in 4 and 37 degrees, respectively. Western blot analysis seen in (**A**) demonstrates the Aß1-42 preparations separated under non-reducing conditions on a 10% Tris-Tricine gel. In (**B**) the left picture demonstrates Aß1-42 preparations separated under reducing condition on a 10% Tris-Tricine gel. The right picture depicts the molecular profile of the Aß1-42 preparations under reducing condition on a 10% Tris-Tricine gel found in the NG2+HOPC cell lysates and supernatants of after 18 h of incubation. (JPEG 322 KB)

Additional file 4: Figure S2: NG2 western blot analysis of immunoprecipitated cell lysates and culture supernatants NG2+HOPC at baseline conditions, and pooled human CSF. +) indicates pre-treatment with chondroitinase ABC (chABC) –) indicates no chABC pre-treatment. The western blot shows that the clone B5 mouse anti NG2 antibody recognized a smear between 300–420 kDa when analyzing cell lysates of unstimulated NG2+HOPCs. Upon treatment of the cell lysate with chondroitinase ABC this smear was absent and only a distinct band at 300 kDa was seen. A smear between approximately 290–420 kDa was detected when examining cell culture supernatants from unstimulated cells and additional bands at approximately 240 kDa were seen. Also this smear was lost after enzymatic treatment and only a band at 290 kDa and a faint band at 240 kDa could be detected. Human CSF yielded two NG2 bands visualized at 290 and 240 kDa using western blot, and the same bands, although fainter, were also seen with enzyme treated human CSF (Figure 4). A similar detection profile was found when rat CSF was analyzed (data not shown). (JPEG 109 KB)

## Abbreviations

APOE ϵ4: Apolipoprotein Eϵ4
AD: Alzheimer’s disease
Aβ: Amyloid beta
BSA: Bovine serum albumin
CNS: Central nervous system
CSF: Cerebrospinal fluid
DMEM: Dulbecco’s modified eagle medium
DMSO: Dimetylsulfoxid
EAE: Experimental autoimmune encephalomyelitis
ECL: Electrochemiluminescence
ELISA: Enzyme-linked immunosorbent assay
GFAP: Glial fibrillary acidic protein
IHSI: Immunohistochemical staining
LDH: Lactate dehydrogenase activity
LPS: Lipopolysaccarides
ML: Molecular layer of hippocampus
MMSE: Mini–mental state examination
MS: Multiple sclerosis
MSD: Mesoscale discovery
MSCP: Melanoma cell surface chondroitin sulfate proteoglycan
ND: Non-demented controls
NFT: Neurofibrillary tangles
NG2: Neuron glia 2
NG2+HOPC: NG2 expressing human oligodendrocyte progenitor cells
OPC: Oligodendrocyte progenitor cells
PBS: Phosphate buffered saline
PMD: Postmortem delay
P-tau: Phosphorylated tau
T-tau: Total tau
WMD: White matter disease.
