# Supplementary figures and images for: NG2 cells, a new trail for Alzheimer’s disease mechanisms?
Source: Acta Neuropathol Commun. 2013 May 9;1(1):7. doi: 10.1186/2051-5960-1-7 (PMC4046664; doi:10.1186/2051-5960-1-7)

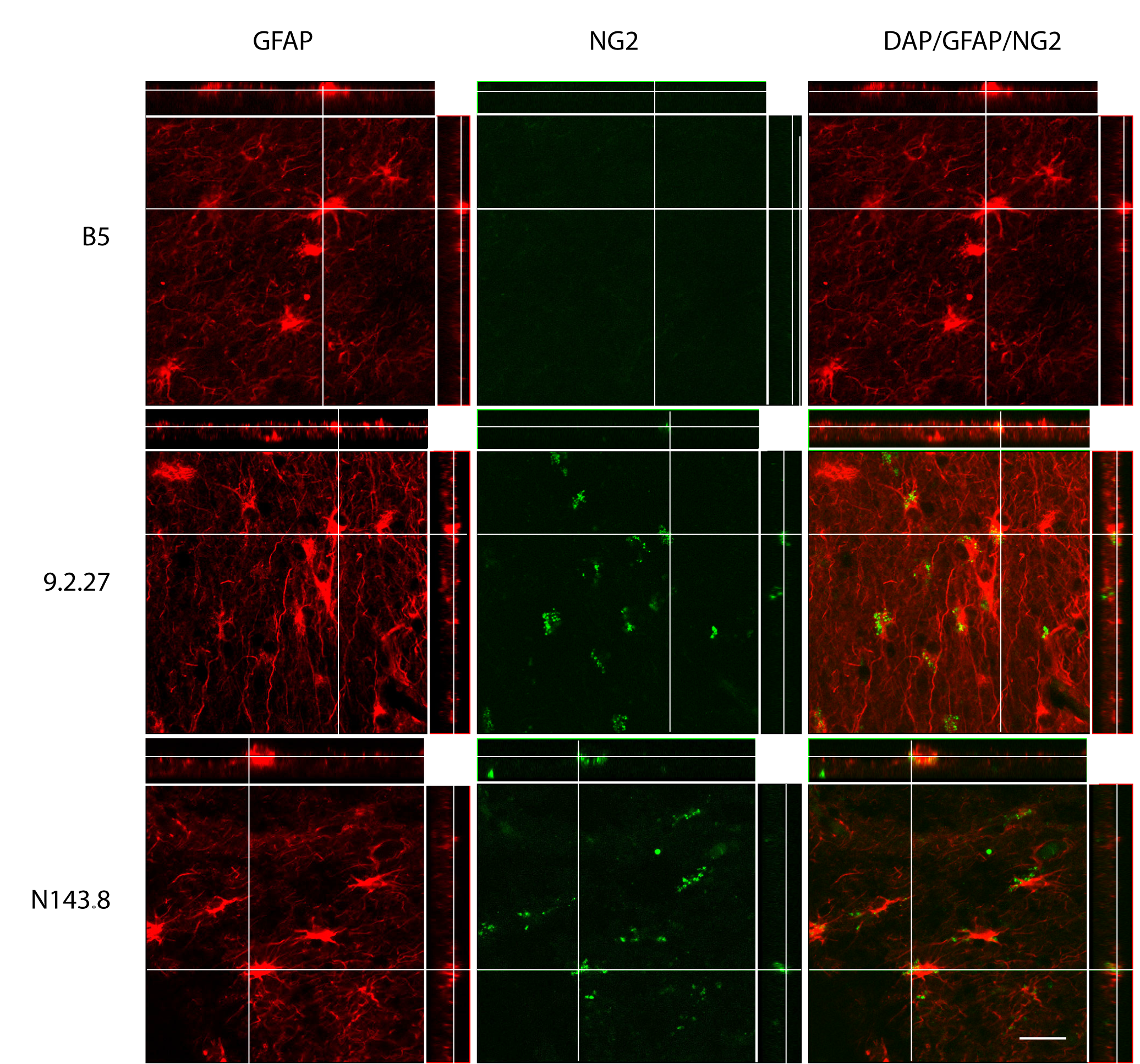

Supplement: Supplementary file 1 — Additional file 1: Figure S3: Confocal images showing the hippocampal molecular layer of an AD patient double stained with the three different NG2 antibody clones B5, 9.2.27 and N143.8 (green) together with GFAP (red). Merged images of the respective NG2 antibody and GFAP are found in the lane far to the right. Scale bar = 25 μm. (TIFF 18 MB) [file 40478_2013_10_MOESM1_ESM.tiff]

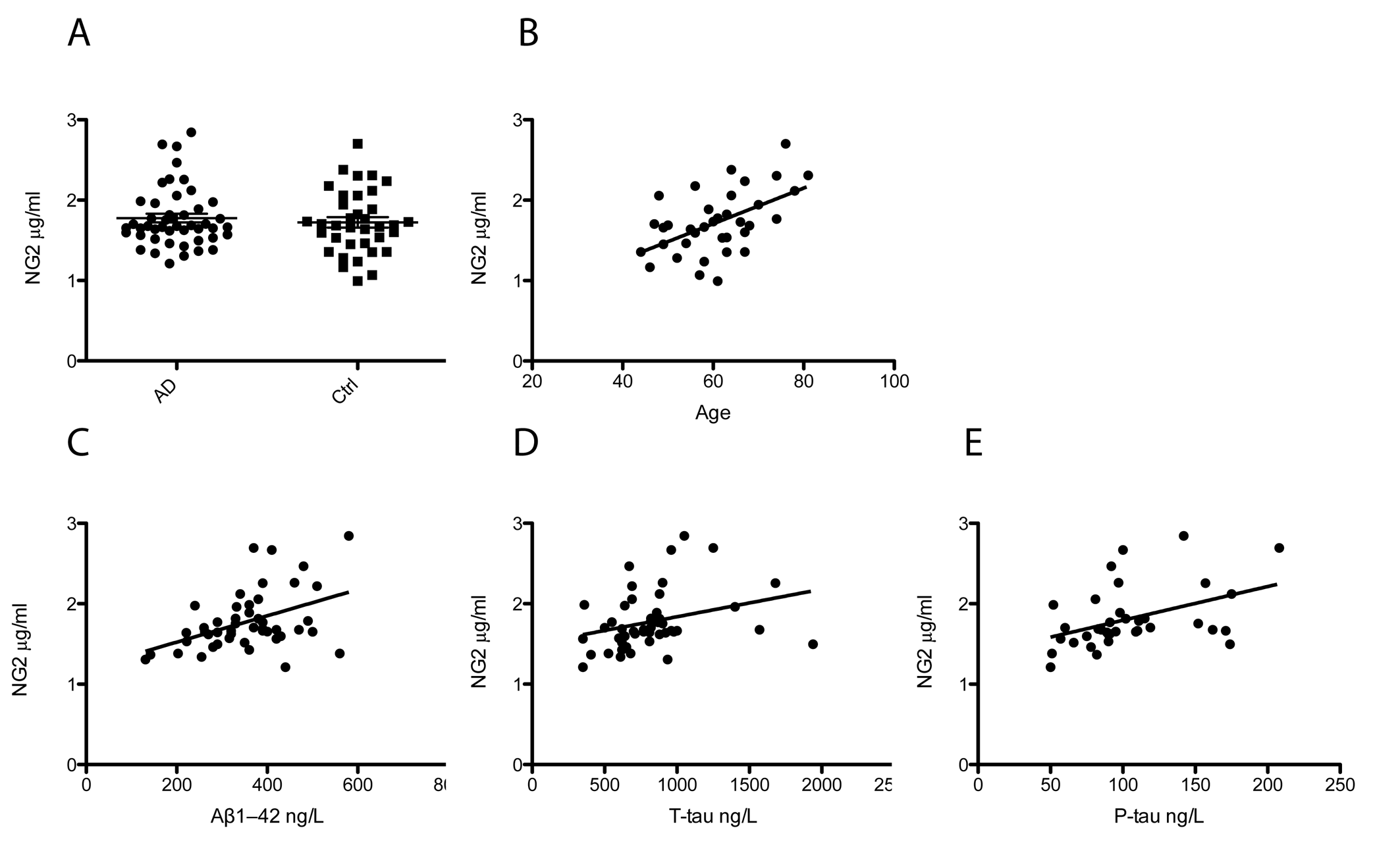

Supplement: Supplementary file 2 — Additional file 2: Figure S4: Plots showing A) Unadjusted NG2 levels in CSF from AD patients and non-demented controls prior to ANCOVA analysis with age as a covariant (AD=Patients with Alzheimers’s disease, Ctrl = non-demented controls). B) NG2 levels in CSF from non-demented controls correlates with age (p=0.001). C) Unadjusted NG2 levels and Aß1–42 levels in CSF from AD patients prior to partial correlation analysis with age as a covariant. D) Unadjusted NG2 levels and Total tau (T-Tau) levels in CSF from AD patients prior to partial correlation analysis with age as a covariant. E) Unadjusted NG2 levels and hyper-phosphorylated tau (P-Tau) levels in CSF from AD patients prior to partial correlation analysis with age as a covariant. (TIFF 8 MB) [file 40478_2013_10_MOESM2_ESM.tiff]

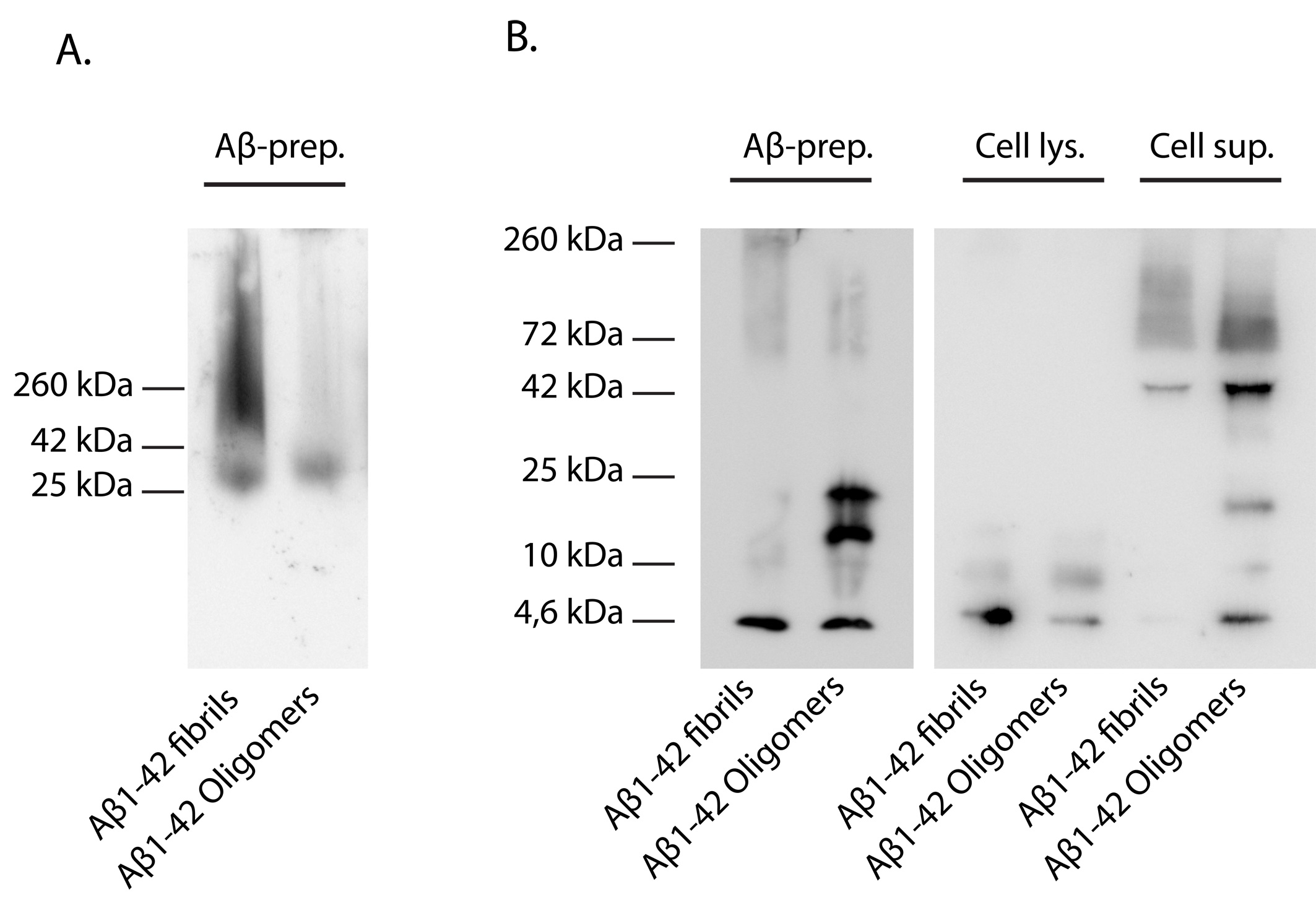

Supplement: Supplementary file 3 — Additional file 3: Figure S1: Oligomer and fibril preparations were incubated for 24 h in 4 and 37 degrees, respectively. Western blot analysis seen in (A) demonstrates the Aß1-42 preparations separated under non-reducing conditions on a 10% Tris-Tricine gel. In (B) the left picture demonstrates Aß1-42 preparations separated under reducing condition on a 10% Tris-Tricine gel. The right picture depicts the molecular profile of the Aß1-42 preparations under reducing condition on a 10% Tris-Tricine gel found in the NG2+HOPC cell lysates and supernatants of after 18 h of incubation. (JPEG 322 KB) [file 40478_2013_10_MOESM3_ESM.jpeg]

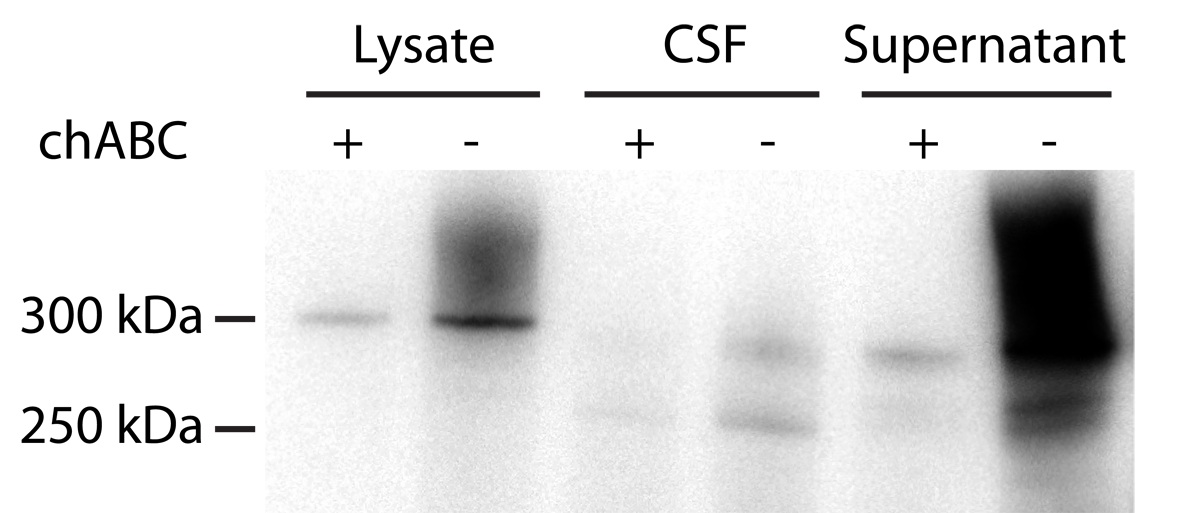

Supplement: Supplementary file 4 — Additional file 4: Figure S2: NG2 western blot analysis of immunoprecipitated cell lysates and culture supernatants NG2+HOPC at baseline conditions, and pooled human CSF. +) indicates pre-treatment with chondroitinase ABC (chABC) –) indicates no chABC pre-treatment. The western blot shows that the clone B5 mouse anti NG2 antibody recognized a smear between 300–420 kDa when analyzing cell lysates of unstimulated NG2+HOPCs. Upon treatment of the cell lysate with chondroitinase ABC this smear was absent and only a distinct band at 300 kDa was seen. A smear between approximately 290–420 kDa was detected when examining cell culture supernatants from unstimulated cells and additional bands at approximately 240 kDa were seen. Also this smear was lost after enzymatic treatment and only a band at 290 kDa and a faint band at 240 kDa could be detected. Human CSF yielded two NG2 bands visualized at 290 and 240 kDa using western blot, and the same bands, although fainter, were also seen with enzyme treated human CSF (Figure 4). A similar detection profile was found when rat CSF was analyzed (data not shown). (JPEG 109 KB) [file 40478_2013_10_MOESM4_ESM.jpeg]
